# Medical Education, with Reasons for Acceptance of the Homœopathic Law and Philosophy as Its Science of Therapeutics*Introductory to the Course of Lectures for 1885–86 in the Woman’s College and Hospital in New York city.

**Published:** 1885-11

**Authors:** P. P. Wells

**Affiliations:** Brooklyn


### HOMŒOPATHIC PHYSICIAN,

A MONTHLY JOURNAL OF MEDICAL SCIENCE.

"If our school ever gives up the strict inductive method of Hahnemann, we are lost, and deserve only to be mentioned as a caricature in the history of medicine."—CONSTANTINE HERING.

Vol. V. NOVEMBER, 1885. No. 11.

### MEDICAL EDUCATION, WITH REASONS FOR ACCEPTANCE OF THE HOMŒOPATHIC LAW AND PHILOSOPHY AS ITS SCIENCE OF THERAPEUTICS.*

P. P. WELLS, M. D., BROOKLYN.

Medical education in its completeness is a compound of knowledges of many sciences. While in its constitution this is very composite, in its objective it is very simple. It is to relieve human pains and sufferings and cure and prevent human sicknesses. A knowledge of whatever helps to the attainment of these may rightfully be incorporated into the sum of whatever has entered into an education of which these are its legitimate objectives. Preventing, relieving, and curing are the functions of the physicians' office, not to pose before the world as expounders of the inexplicable, and masters of all the unknowable in the universe. To be able to explain everything is the function of the sham—to cure is that of the true physician.

To the attainment of this end there have been many ways laid out in preceding generations which were to be followed by those who should cure. These have had their origin in the minds of ingenious men, who imagined the nature of the different factors in the problem of cure, and also the processes by which this was to be effected, and this they called "*theory.*"

* Introductory to the Course of Lectures for 1885–86 in the Woman's College and Hospital in New York city.

There have been many of these theories. Together they constitute the history of practical medicine from its beginning down to the birth of a knowledge of the divine law of therapeutics, the promulgation of which among men was given to Samuel Hahnemann. Previous to this, practical medicine was *theory*—*i. e.*, *imagination*. This has survived the advent of law, and is now all about us in the garb of *Allopathy*. The record of practical medicine from the time of Hahnemann has incorporated into it a new element. Theory has been displaced by *law*. This element it is which characterizes the system he proclaimed to the world and called *Homœopathy*. This has survived the opposition, abuse, and hate of the older—the system of theory—and the two now exist together in the world. *Allopathy*, theory, imagination, guessing, on the one side, and *Homœopathy*, in itself a law, God-given, which declares the relationship between sickness and their curatives, and points the way to their discovery in all curable cases. Here in a single point are the characteristics of the two divisions, in which we meet the practical medicine of to-day—imagination on the one side and law on the other. To which shall we give our adherence and confidence?

It would seem that the question contains in itself its own and only possible answer. And yet, plain as may appear, the superiority of law to speculative guessing, law has not yet all of science and intelligence on its side. So great is the force of tradition, and so much of power is there in its hold on those who have trusted it and have given their lives and their faith to its control, that these have, from the beginning, been found the most violent and persistent opponents of law. Absurd as it may appear, there have been, and still are, many who prefer the guidance and authority of human guessing to that of this only law of therapeutics. This being so, then, when one enters the studies needful to a medical education, he meets at the outset the problem of this education, presenting to him the question of *law* or *theory*. He must make his choice. Let him do this in the light of truth, and do it loyally, firmly, finally. Let there be no insane attempt to mix the two, for this is really impossible. He who attempts this, if he perseveres in this course, is in the end only doomed to a drowning in his own folly.

If we now receive the law of relationship between curing agents and sicknesses as given to us by Hahnemann, what are the grounds of our acceptance of it as truth coming to us with divine authority? What does this law say to us that, as wise seekers of truth, we would present to the ignorant or the skeptical as a justification of our choice?

*First.* It demands, before proceeding to the choice of remedies for pains and sicknesses, an exact knowledge of the *facts* of the case in hand—*facts*, and not our own imaginations or those of other men as to the condition of internal parts or organs in the case, which condition is charged with the responsibility for the sufferings and danger of the case. It demands a knowledge of *all* the facts which can be known, and therefore are knowable, instead of imaginations of supposed conditions of which no man living can know, because while the patient lives these are wholly in the category of the unknowable; and when he is dead the case is but little better, for whatever is learned from autopsies comes too late for practical purposes. This knowledge includes the history of the case as well as that of the facts perceptible to patient and prescriber. Then the law goes farther, and demands of the prescriber that he shall have a like knowledge of all the facts of the action on the organism of the remedy he is to employ, in the same detail as is that of the sickness to be cured. This is the first great distinctive feature of the system of law. It deals only with the knowable, and insists that this shall be known in its entirety, both as to sickness and remedy. In this it stands alone of all which has claimed to represent a system of practical medicine in all past history. And this is the first fact we present which challenges our confidence in the truth of the system of law.

As first propounded by Hahnemann, this system was met and opposed because it ignored the imaginations of the current medicine of the day. "This totality of the symptoms—these knowable facts—and this all? Where then is to be the place of the science of pathology, and what can be the use of it? and what can any system of practical medicine be without this which makes so great a part of that which is our great boast? It is only symptoms. The attempt to base a practical therapeutics on these is only to deal with the surface of things. It is superficial, and only superficial, neglecting to go to the depth of things, as we do, in our imaginations!" These and like objections were and are brought against this system, which demanded facts and a knowledge of them as opposed to that which only proceeded on theories and hypotheses, and held these as not only sufficient, but indispensably necessary, to the duties of the practical prescriber.

In reply to such objections to this system of law, we say, first, when we present it to your confidence, we have no apologies to offer to these objectors, either for the system or its author. They need none. If they are accused of partial views of the sciences necessary to a complete medical education, and neglect of some necessary to an intelligent therapeusis and its practical administration, it is a sufficient reply that Hahnemann and his system require a knowledge of *all* that is *knowable* of the factors of problems of practical healing, and he or that which demands more is only acting the part of ignorance or folly. He who demands more than *all* existing facts is simply insisting on acceptance of his own imaginations being received as of equal authority with facts, or that these be permitted to displace facts, which the system of law will not accede to. In this very point the antagonism of the systems of law and theory has its chief existence. It is not a little singular that human vanity and conceit can go to this extent in opposing a system of law and philosophy and a practice based on these, and bring nothing as a substitute with a foundation more substantial than sheer imagination or hypothesis. And certainly human conceit, arrogance, impudence, and pride can go no farther than it has gone when it claims for this no system of mere fancies that this, and not the system of law, is to be accepted by men as the embodiment of "scientific medicine." And it seems the more strange and singular if we have examined this pretended system, and have seen its emptiness, and the fact that the therapeutics with only a theoretic foundation are as destitute of all belonging to true science as the mummy of Ramesis the First is of life. Truly law owes no apology to theory. It offers none, but instead, asserts its own superior authority and worth with the confidence of a perfect assurance. If any are disposed to discredit the confidence of this assurance, and call it only unseemly "dogmatism," we would remind such an one that a man with truth on his side has a perfect right to dogmatize, as to all who have nothing better to oppose to it than theories and hypotheses, and that dogmatism here is rather graceful than unseemly.

It is not forgotten that there have been and are those who have appeared as apologists for what they regarded as the defects of this system of law. But such have given us no new facts to redeem their alleged defects. They have suggested no improvements which could give added efficacy to the therapeutics of law. Like the habitual carpers at our materia medica, they have only asserted imperfections which they have done nothing to amend. And but little attention to their utterances and movements is necessary to a discovery of the apparent animus and motive of these apologists. Their objective seems to be a harmony and blending of the systems of law and theory, that of the two they may make one, and so have peace. In their efforts for this they have been chiefly busy with efforts to destroy all which is characteristic of the system of law, that it may be more acceptable to theorists, evidently calculating on the ready reception of law, when dead, by those who have in all their history had so little to do with any principles with life in them. These apologists would seem to be ambitious of blending the living with the dead, and would have us believe that the intended result is to be only in the interest of the living. We accept no such apologies. We respect no such apologists. Before all such, and to all such, law may well exclaim—

"*Timeo Danaos, et dona ferentes.*"

Indeed, they bring no gifts, and are expected to bring none, except it may be an acceptance of the cadaver of Homœopathy after its life has been sacrificed by its professed friends. And then, if this be so accepted, which party is the gainer, and what has it gained?

If these apologists have attempted improvements of the system of law at any time, it has been by a sacrifice of the second reason I would present as a claim on your confidence in its truth and authority—viz.: its *simplicity*. It needs no aids from outside sources to secure to its practical administration its greatest possible successes. It accepts none. Wherever these have been thrust into that administration they have only and invariably damaged the record: something from old physic, perhaps to appear to be doing *more* for the sick, and so to relieve the anxiety and satisfy the prejudices of friends. But the *more* from this source has always been the *worse*. Law will have simple obedience, and to this, and this alone, it promises success. The disaster which is so certain to follow violations of law by the introduction of means it neither calls for nor sanctions, has only this one poor consolation for its victims or their friends: "Everything has been done that could be done." Yes; and hence the disaster. It has come because "everything has been done" except the *one* only thing law demanded.

We have said we present the system of law to your confidence because of its simplicity. In this it is like all natural laws. We use the term as applicable to its nature and as opposed to the complex. It is simple in its expression—*Like cures like.* The terms are brief and easily understood, it would seem. But it may be possible to make a mistake in this last, if we hastily conclude that, because simple, therefore easy to be understood. Perhaps, before we thus conclude, if we ask and endeavor to answer the question, "What is the like which cures?" we may find the answer not so easy as we may have been tempted to believe, because of the simplicity of the law. Though the law demands a recognition of *all* the facts of the case, this is not because each fact is of equal importance to every other in disclosing the curative relation to the case of the *one* drug the law demands of us that we find in the discharge of clinical duties under its direction, but because until all are exposed we have no certainty that that which is most important to this discovery has not been left out of sight; and so a failure to find the simillimum is the result, and so the consequent failure to cure. Hence one of the elements of first importance in the education which qualifies one to cure the sick is that knowledge which enables the prescriber to distinguish between those symptoms which are most important as indices to this relationship and those which are less so. To become master of this element is not an easy matter. To become a master in specific prescribing without this knowledge is simply impossible.

The most that can be done for the beginner to aid him in its mastery is to give him general principles for his guidance. Here is one: Those symptoms which belong to a class of sicknesses, and are found in each example of it, and which dominate diagnosis, are of very little importance as indices of the specific curative. As an example, the pains, tenesmus, tormina, etc., of dysentery, without which no case is dysentery, are of first importance to the diagnosis, but are the least helps to the discovery of the curative. It is that which is characteristic of the case in hand, and not that which belongs to every member of the family, which points us to the specific. It is a simillimum to these characteristics of the case which we are to find in the record of some drug, and not to those of the family. The law in this is simple. Its language is, the *one drug*. The duty of finding this, under the guidance of law, is not seldom difficult. In this simplicity it stands in sublime contrast with the medicine of theory, which accepts any and everything the prescriber thinks may do his patient good; as is also the administration of this, singly or in mixture, as the case may be, with the giving of the one specific when found. Here are the two—the one guessing, despairing, giving anything which he imagines will help his case, while the other, with his specific, is able to say, I *know*, because I have the warrant of God's law for my assurance.

Then we present a third reason for our confidence in our law, *viz.: its completeness in itself*, which makes it equal to all needs of sick conditions of men. It is as appropriate to the gravest of these as to the most trifling. To the new and strange, as to the old and familiar. Its practitioner, who is equipped for its practical duties, is as ready for the first case of a strange plague as for any subsequent one. No case of new sickness is a stranger before the law. It, as are all others, is met by the simillimum to its phenomena, and in this is found its master. So that to its completeness in itself, having no needs of outside help, it adds a fourth reason to our acceptance in its *universality of application*—it meets the wants of all curable cases in all lands and climes. This fact alone stamps it as one of nature's laws. And besides this, there is no one of the proposed methods of treating the sick, which does not fail to establish itself as a law, by reason of failure to sustain this character of universality of applicability to all sicknesses. Being a natural law, it is unchangeable. Time has no power to change its relationships or to diminish its efficacy.

This does not mean that diseases called by the same name are always to be cured by the same drug. The system of law has nothing to do with names when it promises cure by the use of the simillimum. In this again it is found in sharp contrast with the medicine of theory, which deals only with *names*, which are accepted as representing *things*. The first duty of the theorist is his diagnosis, *i. e.*, find a name, and then deal with what he understands this name to represent. The prescriber, under the authority of law, disregards names wholly when he is searching for his specific remedy. He is engaged with the phenomena of the sickness before him because it is the simillimum to these which cures. The law does not concern itself with names. Hence it may happen that when called to treat a case—it may be of whooping cough—the remedy which cured all cases last year is of no value in treating what we call by the same name this. The reason is this—though the defining or general symptoms compel the use of the same name, the totality of the symptoms are changed, and this, under law, compels a change of the remedy. Sicknesses change, and so call for change of remedy, and it is one of the glories of our law that it is always ready to meet such changes, and never is surprised by them.

Such is the law we commend to your confidence, as from the omniscience and benevolence of the Supreme—His one great gift to humanity for the relief and cure of its pains and sicknesses; and these are some of the evidences of its divine origin. In one word, its perfect adaptedness to all the needs of relief proclaims its origin, while the triumph of its intelligent administration challenges your confidence in, and loyalty to it, in all your future studies and labors. The medical education which you are now endeavoring to gain is that, and all that, and only that, which contributes to an intelligent administration of this law. It is, if rightfully made up, composed of two parts—one training your own powers of mind into a fitness for this sublime duty, the other of gathering into the mind such knowledge of facts and principles as are involved in this duty.

And, first, of your own mind. And here, from the nature of the case, you must be almost wholly dependent on yourselves. No one can help you much in this. In the initiatory endeavor to prepare the mind for the serious duties of your future life, first, with all your strength and in all time, cultivate your powers of *attention* and *observation*. Insist on seeing, and carefully, whatever is before your eyes, for this is just what your eyes were given to you for. If this seems an easy thing, and one which will do itself, because you cannot but see what is before you, don't make a mistake and fail to cultivate these powers. It is not easy to see all that is before one. Few persons do this, and no one does who has not cultivated the faculty of observation. This, in the practice of specific medicine, is a power of the very highest importance. How are you to cure by finding and giving the remedy which has in its record facts most like those of the case you would cure if you have not seen these facts? If not seen, their necessary comparison with the materia medica record is impossible. Therefore see everything before you. For example, the patient being in bed, note his position. Is he on his back or side? If on the side, on which? Note his posture; is he lying straight or bent? Is he quiet or restless and constantly moving? Is his aspect tranquil or anxious, expressive of pain or peace, of reason or delirium? The color of the face—is it pale or red? If red, dark or bright? Is the red in circumscribed spots or diffused? Is the color dirty gray? Are there dark circles under the eyes? Is the general surface warm, hot, cool, or cold; dry, damp, or wet? If wet, is the perspiration hot or cold? Is the morale tranquil or excited? If excited, what is the character of the excitement? What is the character of the respiration and pulse? These and all other visible facts are to be carefully noted; and the point we wish to make is that you are so to train your power of observation that it will permit no one to escape notice. It is no part of our duty or intention to enter on an explanation of the relation or significance of these facts. This would be to usurp the duty of your teacher, which I would avoid. By a careful training of this faculty you may come to that pass with it which will at a glance give you intelligence as to the nature of the problem before you, and a power to deal with it successfully which those who are careless of this duty can never either emulate or understand.

Next in importance to a cultivated faculty of observation is the possession of the art of gathering *all* the symptoms of a case to be treated, and the first step toward acquiring this art is to get a definite idea of what constitutes a symptom. Do you say, "Why, a symptom is only a simple fact"? This view is wholly defective and inadequate to the expression of this constitution as before the specific prescriber. For him a symptom is much more than this. It is a fact, but not a *simple* fact. So far from this is it, that it is a fact with all that belongs to it of circumstance, condition, and relation to time, and to all the functions of all the organs of the body—how this fact affects each and how each is affected by it. How is it affected by motion or repose—by position or change of position? What are the qualities of this fact? If it be a sensation or a pain—what its exact location—what the time and circumstance of its appearance, aggravation or relief. These and many other elements which complicate the nature of this fact are to be taken into the account before the specific prescriber can accept it as a complete symptom. The reason of this is because the relationship between the sickness and its curative is found in the similarity of these complicating elements to like elements in the record of the materia medica. To gather symptoms, thus understood, is the daily and life duty of the homœopathic prescriber; and until this is accomplished a homœopathic prescription is an impossibility. The pretense of this, without the performance of this first duty, is ever and always a false pretense. This is not only the most important part of the duty of prescribing, but by far the most difficult. It is the most important because until this is successfully accomplished no subsequent step can be taken under our law for the relief or cure of the patient. It is to be done, and done carefully and right, or if otherwise the duty be performed carelessly, or a part of the needful record be wanting, then finding the curing agent may be an impossibility, by reason of the partial statement of one of the elements of the comparison which is to reveal the curative. The likeness to this which represents but *part* of the case may not have the relationship of curative to your case, and the discovery of a simillimum to this, if it be given, can only end in disappointment and failure. The law requires *all*, and will have it, or it gives us no promise of good. Its promise of cure is to that which is most like *all* the facts of the case. To gather only a part, and proceed to prescribe on this, is only of the nature of breach of contract on the part of the prescriber, both as to the law and the best interests of the patient. All of success in practice depends on the thoroughness and completeness of the performance of this duty. Therefore count no labor or careful study given to this, more than its importance demands. Be content with no less than a constant and perpetual endeavor to strengthen your powers in use in this duty, and to increase their familiarity with the processes by which its objective is obtained. Let there be nothing of slip-slop or hap-hazard in its performance, not even for once, for this once may become the beginning of a habit, and the habit once formed, and practical life becomes a wreck, from which there can be no recovery. Cultivate this power to gather symptoms truly and in their entirety till you have mastered the great difficulty, and then you may safely assure yourselves that you have a large part, and a most valuable part, of a practical medical education. This, if you achieve it, remember, is to be a fruit of your own mind-working. Your teacher cannot give this faculty. It must have its origin in your own conviction of its importance, and its growth from the vigorous impulses of your own will. Never cease trying to do this better and more thoroughly, and the end will be, you will find yourselves doing it better and more thoroughly, and your successes will become greater and more assuring and comforting as you continue to strive.

In view of the supreme importance of this duty, may I not hope to be pardoned if I so trespass on the proper ground of your official teacher as to offer some suggestions as to how this securing of this totality of symptoms may be best accomplished? In the first place, write down your facts as you gather them, and you will save the risk of neglecting some if you trust your memory. Have a small, blank note-book always by you, ready for your record. Have some regular, orderly plan of procedure, trusting nothing to chance. It is not a bad one which begins at the head and inquires as to all possible pains, vertigo, and mental and moral symptoms, with all conditions and modalities connected with them. Then follow with the organs of sense, learning all aberrations of functions and pains, with conditions and modalities as before, and proceed through the organism according to the anatomical schema found in the materia medica record, noting all pains, functional disturbances, and their conditions and modalities as before. Then deal in the same manner with the general symptoms, skin, sleep, and fever. Having gone over all this, and thus carefully, you have a basis on which a rational and specific prescription can be founded.

These two indispensable elements of a medical education you are to acquire mainly by the use of your own powers. No one, not even your teachers, can help you much in this work. The most they can do is in the way of advice as to methods of procedure. Attention and observation engages with objectives which are visible. The art of gathering all the symptoms of the case has largely to do with invisibles, the objective of which is to bring them into light and place them in the record which is to be the basis of a legal and rational proceeding for cure. There is and can be no other foundation for this than this record, and hence the necessity for its completeness and perfection. When law insists on the *perfection* of the record, it means it shall have in it no false entries; that in the answers to the needful inquiries the patient is not to be permitted to deceive himself nor his prescriber by whatever of exaggerations or imaginations of his own. It is not to be forgotten when gathering the symptoms of a sickness, that there is occasionally met a weakness of human nature which is ambitious that its own case shall be regarded as an *interesting* one, and to make it so this variety will not hesitate to draw on imagination, and is certain to exaggerate whatever of facts they may disclose. These are most difficult and not pleasant patients to prescribe for. It is to be carefully guarded against that if such patients succeed in deceiving themselves, they shall not deceive the prescriber.

There is a second part of a true medical education, to which the knowledge of others may be made to contribute. We refer to that part of it which includes a knowledge of the facts and laws of life, sick and in health; the philosophy of sick conditions and of their recovery; a knowledge of the nature and action of the means in use for this end, and their rightful administration both as to form and method—in short, whatever enables one to place his problem of cure in the clearest light of law, and in this light helps to its more ready and certain solution, is to be a part of the capital of every one who is to assume the duties of the practical healer. The educational processes he has passed through, presumably, have had for their objectives a general qualification for these duties. He has been taught a knowledge of the law which underlies and sustains the only "*science*" of therapeutics. He has learned its universality of scope and adaptability to the healing of the sicknesses of men. He has made himself familiar with the corollaries of the law which control its administration, and the right management of the means it employs for healings. It is to be presumed he has been taught these knowledges as they are found in the *Organon of Homœopathic Medicine*, and are found nowhere else. It is presumed he has been taught these and also the true nature of sicknesses as there taught, because if not knowing these, whatever else he knows, he is wholly destitute of the knowledges which furnish the specific prescriber for his work. Without a knowledge of these there is no medical education which is worthy of respect or confidence. A knowledge of these principles, and of how to make practical application of them, *is* a medical education in itself. Other knowledges may be required to give completeness to the sum the healer should know. But whatever else he knows, and not this, he is not the possessor of an education which qualifies him to deal specifically with the sicknesses of men. Without these knowledges the would-be healer is but too likely to drift into the habits and methods of the medicine of theory, and there being no great difference between this and the kind of Homœopathy he knows, represents, and practices, he soon falls to dreaming of a possible union of the practical medicine of law and theory—" on a *scientific basis*."

One thing more. We have only considered medical education as exclusively related to the intellect. If it stops here it leaves its votary but partially fitted for his best work. The heart of the healer must also be educated for the duties of his office. And we would begin this by enforcing the apostolic injunction, "Let patience have her perfect work." In this we do not forget the beauty and need of gentleness and affectionate interest in the subjects of professional care. These in woman are known to be present as a spontaneity. But we begin with patience because of the great difficulty often experienced in its exercise, and because the practice of it is indispensable to the successes an observance of the demands of law promises to us. We inculcate patience first, because often, in dealing with important sicknesses, success will depend so much on its presence. Patience first in gathering the basis of a rational prescription, which is often very difficult. And there are many reasons for this. Some belong to the patient and some to the prescriber. The patient may be of that eccentric order who think it is smart to deceive, conceal, or mislead the inquirer after his symptoms. In this his eccentric vanity presents him to himself as the superior of his doctor, and he likes the view. I have seen and dealt with some of this class, and confess that a proper dealing with them requires a sublimity of patience almost beyond the possible. But this poor vanity is to be borne with, and by persistent perseverance is to be overcome. The truth must be had. Then another class is met who are almost wholly incapable of giving an intelligent account of the elements of their sicknesses. They only know they feel bad, and any attempt at an analysis of their bad feelings is something beyond their comprehension. A pain is a pain with them, and all are alike, and their whole idea of it is limited to the sense of discomfort it causes. These may not be foolishly vain, but they are almost incorrigibly dull, and truth, which must be had, is only obtained from them by utmost patience and perseverance. Each patient will have his personal peculiarities and each present his peculiar difficulties to be overcome, and each will demand more or less carefulness and patience for their mastery. No doubt it is easier to generalize and guess than to strive and overcome the many difficulties incident to a specific dealing with sicknesses. But remember, if you yield to the temptation, and substitute generalization for the individualization a practice founded on law requires, you have by so doing abandoned Homœopathy, and have no longer a right to its honorable name. If for ease's sake, or for any other cause, you proceed to find and give a remedy for pneumonia, for example, instead of searching out the facts of that sickness and finding the remedy for these, as the law requires, you will not only have abandoned Homœopathy, but will have added, probably, to the long list of fatal cases which have been so treated before. It is easier to prescribe for a *name* than for the "totality of the symptoms"—*i. e.*, for all that is knowable of a case; but certainly we know no other reason for this stupid resort. It may be true, as was said recently by a so-called homœopathist of New York, that those in your city "who prescribe on symptoms could be counted on his fingers." If so, then all that follows from the fact is, that the number of those here who are really true homœopathists are all found in this limited number. The doctor who had made this interesting discovery gave the exponent of his own professional character when he answered the question, in a professional consultation, "What are the reasons which decide your choice of the remedy you recommend in the case?" His answer was, "It is Dr. ——'s great remedy in these cases!" His professional life has evidently been an imitation of the child's game, known as "follow my leader." You may meet such—and it is to be feared there are but too many of them. If you do, they may tax you for all the patience you have, and perhaps more.

But there is another field in which you may be called on for your whole stock of patience. You have studied your case, gathered its facts, and, as you believe, have found your simillimum. You have given this, and when the time comes that you look for response in the relief of your patient, it has not appeared. What shall you do? Change your remedy? Not so. Revise your examination of your case and your comparison of its record with that of the materia medica, and if you find your selection justified—wait! "Not able to wait for a thing"—said that great master of prescribing, the elder Gross—"is the original sin." Or, more literally translated, the parent of *all* sins. And in therapeutics there is in this more of truth than rhetoric.

We would emphasize this duty of waiting, because just for the want of this many lives are lost. Let it be remembered, different patients respond to their specific in times which differ greatly as to extent. The same is true also as to the time required by different remedies for their reactive and curative action. Some are reacted on instantaneously, sometimes, and always, if at all, in a short time after their administration. Others only after hours, days, or even weeks. I have seen the best of results, in gravest sicknesses, appear after two weeks of painful waiting. I have seen the worst of results follow the change of remedy which impatience had demanded and practiced, because the expected curative action was delayed beyond the time in which it had been looked for. For the government of practice in the matter of change of remedy and repetition of dose—study the directions of the Master in his *Organon*, obey these implicitly, and then—let patience and your specific have their perfect work.

